# Antithyroid Peroxidase Antibodies in Multinodular Hashimoto's Thyroiditis Indicate a Variant Etiology

**DOI:** 10.1155/2019/4892329

**Published:** 2019-07-21

**Authors:** Pabithadevi B. Mehanathan, R. Raskin Erusan, K. Shantaraman, S. M. Kannan

**Affiliations:** ^1^General Surgery, Tirunelveli Medical College, Tirunelveli, India; ^2^Multi Disciplinary Research Unit, Tirunelveli Medical College, Tirunelveli, India; ^3^Department of Pathology, Tirunelveli Medical College, Tirunelveli, India; ^4^Tirunelveli Medical College, Tirunelveli, India

## Abstract

**Introduction:**

Hashimoto's thyroiditis (HT) is a common autoimmune thyroid disorder, which predominantly presents as a diffuse goiter, with few studies which report HT presenting as multinodular goiter, with variable frequencies ranging from 59% to 78.6% especially from south Indian populations. This variant clinical presentation may have diagnostic challenges which require further analysis. Anti-TPO antibodies are more common (90-95%) in Hashimoto's thyroiditis than anti-TG antibodies in Hashimoto's thyroiditis. This study analyzes the clinical features and the correlation of anti-TPO levels with diffuse and multinodular forms of HT.

**Material and Methods:**

This study was conducted in the Department of General Surgery in a tertiary care hospital in south Tamil Nadu. Patients presenting with clinical features of a thyroid disorder were interviewed and given a detailed clinical, radiological examination and guided FNAC. Those patients diagnosed by FNAC as HT were registered and a sample of 3cc of blood was drawn for T3, T4, TSH, and anti-TPO analysis. All the data were tabulated.

**Results and Discussion:**

Of the 212 patients who presented with goiters, 96 were diagnosed by FNAC as having a cytological picture suggestive of Hashimoto's thyroiditis. Of these 96 patients with HT, 46 (47.9%) were multinodular (HT-MNG), 14 (14.58%) were solitary nodules (HT-SNT), and the remaining 36 (37.5%) were diffuse goiters (HT-D). Of the 46 patients who are HT-MNG, 36.9% had elevated anti-TPO-Ab (more than 35.0U/l) and 63.1% had normal/lower values (less than 35.0U/l). But of 36 patients with HT-D, 77.7% had elevated anti-TPO-Ab levels (>35U/l). Chi square statistics was 15.8346 and the p value is 0.0005 (<.05). Eight cases of HT-D and 3 cases of HT-MNG had hyperthyroidism and 3 cases of HT-D had hypothyroidism and all other cases were in euthyroid state.

**Conclusion:**

Patients presenting as multinodular Hashimoto's thyroiditis have low prevalence of elevated anti-TPO-Ab than diffuse HT which suggests that multinodular form of Hashimoto's thyroiditis is a unique clinical entity with etiopathogenesis that is at variance with the diffuse form.

## 1. Background

Hashimoto's thyroiditis (HT) is a common autoimmune thyroid disorder, characterised by follicular lymphocytic infiltration in the thyroid gland with formation of germinal centers, Hurthle cell change, atrophy of the follicular epithelial cells, and gradual fibrous replacement of the thyroid parenchyma. HT predominantly presents as a diffuse goiter [1], with few reports of a presentation as a multinodular goiter [2]. These multinodular HT patients are reported with variable frequencies ranging from 59% to 78.6% especially from south Indian populations [3]. This variant clinical presentation may have diagnostic challenges which require further analysis.

The destruction of thyroid cells in HT is associated with various cellular and antibody mediated immune processes which include thyroid autoantibodies (TAbs) against thyroid peroxidase (TPO) and thyroglobulin (Tg). Anti-TPO antibodies are more common than anti-Tg antibodies and more indicative for thyroid disease [4] and are detected in 90–95% of AITD patients, 80% of GD, and 10–15% of non-AITD patients [5]. While anti-TPO antibodies may act cytotoxic on thyrocytes in HT they do not have an established role in GD [6]. Exact values cannot be compared directly since sensitivities of the assays differ, but the range of >35 U/l for anti-TPO, according to most laboratories can serve as approximate indication for the diagnosis of HT [7]. Various studies reported a high occurrence (61%) of Hashimoto's thyroiditis in the form of diffuse goiter and 93 percent of these patients were anti-TPO positive [8]. Similar studies on multinodular type of HT are sparse and hence its etiopathogenesis requires to be documented. This study analyzes the clinical features and the correlation of anti-TPO levels with diffuse and multinodular forms of HT.

## 2. Methodology

This study was conducted in the department of General Surgery in a tertiary care hospital in south Tamil Nadu after obtaining the institutional ethics committee permission. Patients presenting in the outpatient department with visible thyroid enlargement or with clinical features indicative of a thyroid disorder were interviewed and given a detailed clinical examination, which was followed by radiological examination with ultra sonogram, biochemical tests for thyroid hormones, and guided fine needle aspiration cytology (FNAC). The patients were diagnosed to have Hashimoto's thyroiditis by FNAC based on the presence of oxyphilic (Hurthle) cells, lymphocytes, few plasma cells, and the presence of moderate to scant amount of colloid in the background.

These patients diagnosed by FNAC as HT were registered and a sample of 3cc of blood was drawn for the thyroid analysis (T3, T4, TSH, and anti-TPO) which were performed using chemiluminescence immunoassay (Beckman & Coulter Inc.) using Access II kits and calibrators after obtaining a written informed consent. These patients were then prescribed medical and surgical management as per the standard protocols, and thyroidectomy was performed when indicated. The thyroidectomy samples were sent for histopathological examination and only those patients diagnosed as Hashimoto's thyroiditis in histology were included for final analysis in the study.

The patients with histological diagnosis of HT were classified as those presenting as diffuse goiter (HT-D), solitary nodule of thyroid (HT-SNT), or multinodular goiter (HT-MNG) based on the clinical and radiological evidences. A structured proforma was used to document the data of clinical history, present and past morbidities, and socioeconomic, demographic, clinical, radiological, cytopathological, and biochemical parameters including serum T3, T4, TSH, and anti-TPO antibody. The results were tabulated and analyzed using a chi square test.

## 3. Results

Of the 212 patients who presented with goiters, 96 were diagnosed by FNAC as having a cytological picture suggestive of Hashimoto's thyroiditis. Of these 96 patients, 91 underwent surgical management with hemi/subtotal thyroidectomy. The tissue samples of all these 91 patients presented with histological features confirmatory of HT including dense lymphocytic infiltrates forming follicles with reactive germinal centers and Hurthle cell change of the follicular epithelial cells. Of these 96 patients with HT, 46 (47.9%) were multinodular (HT-MNG), 14 (14.58%) were solitary nodules (HT-SNT), and the remaining 36 (37.5%) were diffuse goiters (HT-D).

The assays for T3, T4, TSH, and anti-TPO antibody are tabulated with the clinical information. Of the 46 patients who are HT-MNG, 17 had elevated anti-TPO-Ab (more than 35.0U/l), and 29 had normal/lower values of less than 35.0U/l. Of 36 patients with HT-D, 28 had anti-TPO-Ab levels >35U/l and 8 patients had anti-TPO-Ab levels <35U/l (Table 1). Chi square statistics was 15.8346 and the p value is 0.0005(<.05). Eight cases of HT-D and 3 cases of HT-MNG had hyperthyroidism and 3 cases of HT-D had hypothyroidism and all other cases were in euthyroid state.

## 4. Discussion

Hashimoto disease is a complex autoimmune disease, more prevalent among elderly women [9], and in geographic regions which are iodine sufficient (e.g., USA) or iodine excess (e.g., Japan) [10]. Thyroid peroxidase is a transmembrane protein located in the apical membrane of thyroid follicular cells, involved in the synthesis of thyroid hormones. Thyroid microsomal antibodies, namely, anti-TPO antibody, is a thyroid autoantibody targeting thyroid peroxidases [11] and is considered diagnostic of both Grave's disease and Hashimoto's thyroiditis, while in Hashimoto's thyroiditis, anti-TPO antibody is reported in approximately 90% of patients [12].

Our study conducted in south Tamil Nadu has observed that 47.9% of our patients with Hashimoto's thyroiditis were having multinodular thyroid, while 14.5% were solitary nodules and 37.5% were diffuse forms. Of the HT patients with diffuse thyroid enlargement, 77.7% had elevated titres of anti-TPO-Ab, while 36.9% of our patients with multinodular goiters had elevated titres of anti-TPO-Ab and 63% had low titres of anti-TPO-Ab. But the histological patterns of multinodular and diffuse forms of HT were not different.

Two studies reported from southern India, Tina Thomas et al. (2014), reported that 61% of Hashimoto's thyroiditis presented as diffuse goiter with 93% of them positive for anti-TPO-Ab [8], while Anila KR (2016) reported that nodular form of HT was seen early in the progression of disease, associated with normal TSH and high anti-TPO levels, which became predominantly diffuse later [13]. Shirish.S.C et al. (2014) reported that 67.30% of their patients had diffuse goiters, while 30.76% had uneven enlargement and 1.92% had solitary nodule, while serum anti-TPO-Ab were elevated in 61.52% patients [14]. These studies are at variance with our observation that nodular forms of HT were associated with higher incidence of high titres of anti-TPO-Ab, while we observed in the converse that it was lesser in our patients. We have also reported the analysis of clinical data of Hashimoto's thyroiditis patients for a period of 3 years (2013-2015) which indicated a predominance of multinodular HT with euthyroid state in this region at variance with western literature [3].

In routine, Hashimoto's thyroiditis is diagnosed by the elevated level of anti-TPO-Ab and high level of TSH and treated with levothyroxine, and many studies [15] have shown that the serum levels of anti-TPO-Ab decline during levothyroxine treatment. But in our patients with multinodular form of HT with low titre of anti-TPO-Ab, the established conservative line of treatment is ineffective.

Several population-based studies [16–18] have reported higher prevalence of anti-TPO antibody in regions with higher iodine consumption, namely, 18% in iodine sufficient areas [19] and 25% in areas with excessive iodine intake [20], while that was approximately 13% in iodine deficiency areas [16]. There are reports of almost fourfold increase in anti-TPO positive HT after exposure to higher iodine levels consequent to the iodine prophylaxis in iodine deficient areas [19–21]. Katja Zaletel et al. (2011) have reported [22] that deliberate exposure to 500 *μ*g of iodine provoked thyroid autoimmunity synthesis in 20% of previously healthy individuals [23]. In a study conducted at Denmark also reports that the prevalence of TPO-Ab before and after mandatory iodization of salt was 14.3% and 23.8%, respectively [24]. The familial clustering of autoimmune thyroid disease and the presence of anti-TPO-Ab in 16.7% to 60% of first-degree relatives of patients has been reported by several studies [25–29]. Among first-degree relatives of patients with HT, 34% were demonstrated to be positive for anti-TPO-Ab compared to 13% among persons not related [29]. The sibling risk ratio for HT, calculated on the basis of the data from the NHANES III study, was 28, confirming a significant interaction of genetic factors in disease development [30]. In Hashimoto's thyroiditis, however a small subset of patients (10%) are reported with clinically evident disease, and a negative serum anti-TPO-Ab [31].

This study suggests that multinodular form of Hashimoto's thyroiditis is a unique clinical entity with etiopathogenesis that is at variance with the diffuse form, with possible genetic variations and differences of metabolic and environmental factors. Since our sample size is small, the preposition of multinodular HT as a unique clinical entity with specific etiopathogenesis needs further study.

## 5. Conclusion

Multinodular form of Hashimoto's thyroiditis is a unique clinical entity with possible specific etiology and pathogenesis, different from the established etiopathogenesis attributed to the diffuse form of HT.

## Figures and Tables

**Table 1:** Distribution of HT and relation with thyroid hormone status and anti-TPO Ab.

No	Parameter		HT-D	HT-SN	HT-MN	Total
1	No. of Patients (Percentage)		36(37.5)	14(14.58)	46(47.92)	96

2	Mean Age in years		41.6	44	43	42.87

3	Gender	Male	1	1	0	2
		Female	35	13	46	94

4	Presenting Features	Goitre	35	14	46	96
		Hypothyroidism	0	0	0	0
		Hyperthyroidism	1	0	0	1

5	Biochemical Tests					
	*Serum T3*	High	8	0	3	11
	(Ref Range: 60-200 ng/ml)	Normal	25	14	43	82
		Low	3	0	0	3
	*Serum T4 *	High	8	0	3	11
	(Ref Range: 4.5–12ug/ml)	Normal	25	14	43	82
		Low	3	0	0	3
	*Serum TSH*	High	3	0	0	3
	(Ref Range: 0.3-5 uIU/ml)	Normal	25	14	43	82
		Low	8	0	3	11
	*Serum Anti-TPOAb *	<35	8	10	29	47
	(Ref Range: 0.25-35 U/l)	36-200	4	0	6	10
		201-500	5	2	3	10
		501-976	8	2	4	14
		>976	11	0	4	15

*Note.* HT: Hashimoto's thyroiditis, HT-D: diffuse type, HT-SN: solitary nodular type, and HT-MN: multinodular type.

## Data Availability

The data used to support the findings of this study are available from the corresponding author upon request.
